# Colonic atresia and anorectal malformation in a Haitian patient: a case study of rare diseases

**DOI:** 10.1186/2193-1801-3-203

**Published:** 2014-04-26

**Authors:** Max Herby Derenoncourt, Gerard Baltazar, Tamar Lubell, Alice Ruscica, Cyril Sahyoun, Francisca Velcek

**Affiliations:** Hopital Bernard Mevs, Universite Notre Dame d’Haiti, Port-au-Prince, Haiti; Morgan Stanley Children’s Hospital of New York, Columbia University Medical Center, New York, NY USA; Downstate Medical Center, State University of New York, Brooklyn, NY USA

**Keywords:** Colonic atresia, Anorectal malformation, Intestinal atresia, Wingspread classification

## Abstract

**Introduction:**

Colonic atresia and anorectal malformation are rare congenital anomalies individually. Few reports of the conditions combined in a single patient have been published in the literature. Neither colonic atresia, anorectal malformation or a combination of the disorders has previously been reported in the Haitian population.

**Case presentation:**

A 5-day-old female presented with feculent emesis, failure to pass stool since birth and an imperforate and stenotic anus. Exploratory laparotomy revealed colorectal atresia distal to a malformed cecum and a Wingspread low subtype anorectal malformation without any associated urogenital fistulae. Temporizing percutaneous ileal drainage was followed by second-stage anal perforation and dilation, ileal J-pouch and pull through.

**Discussion:**

This is the first reported case of colonic atresia, anorectal malformation or the combination of the disorders among the Haitian population and one of only a handful of such cases reported worldwide. Although vascular accidents *in utero* have been implicated as the etiology of colonic atresia, simultaneous presence of anorectal malformation suggests a multifactorial cause. Investigation for multisystem abnormalities is warranted. Two-staged operative correction is considered the best treatment; however, long-term postoperative outcomes are uncertain.

**Conclusion:**

The coexistence of colonic atresia and anorectal malformation is a very rare occurrence and presents unique clinical and operative challenges. Investigation for additional congenital abnormalities is appropriate, and although two-stage operative correction is considered the best treatment, long-term outcomes are uncertain.

## Background

Colonic atresia (CA) is a congenital anomaly in which part of the colon has not formed *in utero*. CA represents 5–15% of all intestinal atresias, with an incidence as low as 1 in 66,000 live births (Davenport et al. 1990;Mansoor et al. 2010). Anorectal malformation (ARM) is a developmental defect of the anus and distal rectum and occurs in approximately 1 in 10,000 live births (Nazer et al. 2000).

Worldwide, coexistence of these anomalies in a single patient has been reported only a handful of times (Goodwin et al. 2006;Petropoulos et al. 2004;Dickinson 1967). To our knowledge, neither CA, ARM nor a combination of the disorders has been reported in the Haitian population or greater Caribbean.

Combinations of these anomalies require multi-stage operations and extensive medical management to establish bowel continuity, optimal intestinal absorption and unimpaired defecation. Separately, each may result in profound morbidity, financial burden or mortality (Etensel et al. 2005;Levitt and Peña 2007). In combination, long-term outcome of these anomalies is unclear.

Herein, we describe a case of CA and ARM, coexisting in a Haitian female newborn. We then provide a review of similar cases reported in the literature and discuss possible causes and treatment options.

## Case description

A 5 day-old female presented to a field clinic, suffering from feculent emesis and failure to pass stool since birth. The patient’s mother did not receive prenatal care and had a presumed full-term vaginal delivery at home.

Before transfer to a hospital, the child was treated with intravenous fluid resuscitation and orogastric tube decompression. Feculent drainage from her orogastric tube was noted.

In hospital, the patient presented with malnourishment (2.5 kg, 3^rd^ percentile for age) and moderate dehydration. Her abdomen was grossly distended without signs of peritonitis.

Because external genitalia appeared anatomically correct; the anus was imperforate; and by using a probe, we could distinguish a stenotic anal canal, we classified the child as Wingspread low subtype ARM (Wakhlu 1995).

Abdominal radiograph demonstrated extensive bowel dilatation. Attempt at barium enema revealed no recto-anal fistula.

Cardiovascular examination revealed no evidence of cardiac anomalies with no murmurs, equal femoral pulses and appropriate pre- and post-ductal oxygen saturations.

Electrolytic and fluid balance was optimized for surgical intervention and parenteral antibiotics were administered.

Exploratory laparotomy revealed grossly intact gastrointestinal system from proximal stomach to patent ileocecal valve. The length of the small bowel was grossly adequate for nutrient absorption.

**Figure 1:**
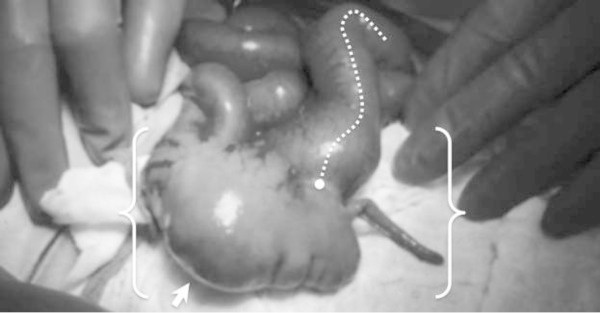
**Colonic atresia.** Terminal ileum (curved, dashed line); ileocecal valve (circle); 5-cm colon segment with appendix (brackets); blind-end (arrow).

However, distal to the ileocecal valve, five centimeters of a blind-ending colon existed with a fully-formed appendix and some bulbous anatomical features of a cecum. But there was no ascending, transverse, descending or sigmoid colon. We visualized the superior mesenteric artery but were unable to visualize the inferior mesenteric vessels. The colonic segment was not attached to the posterior abdominal wall and floated freely in the abdominal cavity (Figure 1).

There was no rectum, no evidence of no intestinal-urinary or genitor-urinary fistulae and no gross anal musculature atresia—the pelvic bowl was an empty cul-de-sac at the base of which, the imperforate, stenotic anus could be palpated, confirming Wingspread low subtype ARM. There was no evidence of malrotation, other urogenital abnormality or abdominal wall defect.

A percutaneous drainage tube was placed into the colonic segment and through the ileocecal valve into the terminal ileum. Post-operatively, the patient remained hemodynamically stable and received enteral nutritional support for two months.

She then underwent a second-stage exploratory laparotomy, J-pouch creation, perforation and dilation of the anal opening and anal pull-through.

The patient recovered from the second-stage operation without complications and is defecating effectively with plans for long-term monitoring and follow-up.

## Discussion

Coexistence of CA and ARM has been reported only a handful of times in the literature (Goodwin et al. 2006;Petropoulos et al. 2004;Dickinson 1967). We present the first case of a combination of the disorders in Haiti and greater Caribbean.

Vascular accidents occurring *in utero* are most commonly implicated in the etiology of CA; whereas, the etiology of ARM is widely considered multifactorial and may include participation of recessive genes (Nazer et al. 2000;Mo et al. 2001;Shinha et al. 2008). Maternal history of first trimester menorrhagia and consanguinity have proven to be risk factors for ARM (Shinha et al. 2008). Prenatal care has proven helpful in prevention and early detection of both these congenital anomalies (Haeusler et al. 2002).

Our patient’s mother did not smoke or use legal or illicit vasoactive drugs of any kind and had no family history of congenital anomalies. She denied first trimester menorrhagia and consanguinity. An impoverished woman, she had no access to adequate nutrition or prenatal medical care. As with previous cases of combined CA and ARM, etiology of our case remains unclear and is considered multifactorial.

Presentation of CA can be delayed by a number of factors, including restricted access to care and lack of adequate diagnostic tools or clinical experience with the disorder (Levard and Boureau 1990). Presentation of ARM depends on the subtype with lower subtypes often presenting with some ability to pass feces (Wakhlu 1995). Signs and symptoms of bowel obstruction are typically present in higher subtypes and may be complicated by sepsis and neonatal peritonitis (Levard and Boureau 1990).

Presentation of the combined disorder of CA and ARM is unclear. However, as in our case, the classical features of bowel obstruction in neonates should raise suspicion for either or both underlying causes. Cardinal signs and symptoms of neonatal bowel obstruction include maternal polyhydramnios, bilious emesis, failure to pass meconium on first day of life and abdominal distension (Juang and Snyder 2012). High level of clinical suspicion supported by basic imaging studies may assist in early diagnosis. Imaging studies performed for the evaluation of patients with colonic atresia include abdominal radiograph and contrast enema.

Our patient’s abdominal radiograph revealed distended bowel loops consistent with intestinal obstruction. Although she was hemodynamically stable, the severity of imaging studies and obvious imperforate anus necessitated urgent operative intervention.

Laparotomy revealed CA and confirmed Wingspread low subtype ARM. While Wingspread high subtype ARM may involve rectal atresia, our case had no evidence of fistulae, presacral mass or anal musculature atresia also commonly associated with high Wingspread classification (Hamrick et al. 2012;Osifo et al. 2014). This fact implies a multifactorial cause of the deformities, with rectal atresia occurring distinctly from anal malformation. An *in utero* vascular accident of the inferior mesenteric artery may help explain the extensive CA; whereas, the ARM may be related to a genetic failure of anus formation.

Optimal surgical correction of combined CA and ARM is two-stage with an ostomy for decompression and evacuation of bowel content, followed by nutritional optimization before definitive correction (Wakhlu 1995;Karmak et al. 2001). Depending on the length of formed colon, definitive correction of CA includes end-to-end anastomosis or j-pouch and pull-through for shorter segments or absent colon (Davenport et al. 1990;Karmak et al. 2001). In our case, the 5-cm colonic segment was malformed therefore was excised rather than used for pull-through. The recommended operative approach to ARM (depending on Wingspread subtype) is to perform a posterior sagittal anorectoplasty versus anoplasty and dilation only if low subtype as in our case (Wakhlu 1995).

In a stable child with CA but without other abnormalities, complete recovery of bowel function can be expected (Mansoor et al. 2010). Wakhlu suggests that after correction for ARM, most patients should be able to achieve normal anorectal function (Wakhlu 1995). Longterm surveillance of the patient’s growth and bowel function is essential (Nazer et al. 2000;Karmak et al. 2001). There are inadequate reports of long-term follow-up of patients with combined CA and ARM; we plan to follow-up and report on our patient’s long-term progress.

## Conclusion

The coexistence of CA and ARM is a very rare occurrence and presents unique clinical and operative challenges. Investigation for additional congenital abnormalities is appropriate, and although multi-stage operative correction is considered the best treatment, long-term outcomes are uncertain.

## Consent

Written consent was obtained from the patient’s mother for the publication of this report and any accompanying images.

## Abbreviations

CA: Colonic atresia
ARM: Anorectal malformation.
